# Bird community effects on avian malaria infections

**DOI:** 10.1038/s41598-023-38660-2

**Published:** 2023-07-19

**Authors:** Juliana Tamayo-Quintero, Josué Martínez-de la Puente, Miriam San-José, Catalina González-Quevedo, Héctor F. Rivera-Gutiérrez

**Affiliations:** 1 Grupo de Ecología y Evolución de Vertebrados, Instituto de Biología, Facultad de Ciencias Exactas y Naturales, Universidad de Antioquia, Medellín, Colombia; 2 Departamento de Parasitología, Facultad de Farmacia, Universidad de Granada, Granada, España; 3 Charles Darwin Foundation, Charles Darwin Research Station, Puerto Ayora, Ecuador

**Keywords:** Biodiversity, Ecological epidemiology, Malaria

## Abstract

In community assembly processes, interspecific interactions play an important role in shaping community diversity, especially at the local scale. Changes in species richness or abundance can modify local infectious disease dynamics, either reducing or increasing the risk of transmission within the community. This study evaluates the effects of bird community on avian haemosporidians infections in a Neotropical region. Bird samples were collected from areas surrounding three dams, and molecular analysis were performed to identify blood-parasitic haemosporidia infecting the birds. Generalized linear models were used to analyze the relationships between the bird community and the prevalence, number of infections, and richness of avian haemosporidian lineages. Non-significant effects of bird community dominance and richness on the prevalence of avian parasites and the number of infections of *Haemoproteus* were found. However, there was evidence of an amplification effect. Host dominance was associated with the total number of infections, the number *Plasmodium* infections and the expected richness of *Plasmodium* lineages, while the expected richness of *Haemoproteus* lineages was associated with the richness of bird species. These findings highlight the role of host community dominance and richness in the dynamics of parasite infections, potentially influenced by the availability of competent hosts. This study contributes significantly to our understanding of blood parasite diversity in tropical birds within a relatively understudied region of South America.

## Introduction

Understanding how patterns in community diversity patterns emerge is one of the most significant challenges in ecology^1^, as it requires going beyond the simple counting of species^2^. In the community assembly process, factors such as interspecific interactions, habitat availability, and heterogeneity have been proposed as the major determinants shaping species abundance and distribution^1,3^. However, most studies on ecological interactions mainly focus on competition and predation as the key drivers of species diversity, while parasitism has received comparatively less attention^4,5^. This is particularly important considering the abundance, ubiquity, and extraordinary diversity of parasites, which can have an impact on animal diversity comparable to that of predators. Host-parasite interactions represent a selective pressure that maintains genetic variability in host populations. Therefore, characterizing the structure of parasite communities and their environmental and biotic determinants is crucial for understanding the diversity and the functioning of ecosystems^4,6,7^.

One of the most studied groups of parasites in ecology and evolution is the blood parasites belonging to the order Haemosporida. This order includes the avian malaria parasites of the genus *Plasmodium* and the related genera *Haemoproteus* and *Leucocytozoon*^8^. These three genera have similar life cycles, being transmitted by hematophagous invertebrates, although the groups involved in their transmission differ. In general, the vectors of these parasites have a cosmopolitan distribution, being absent in few areas of the planet, such as Antarctica^9^. The high diversity of parasites in avian populations and the availability of molecular methods for their detection and identification^8^, make avian malaria parasites excellent models for the study of host-parasite interactions^10^. Different studies have found detrimental effects of these parasites on the reproductive success and survival probability of birds^11,12^. This is especially relevant in immunologically naïve species^13^ as in the case of the introduction of *Plasmodium relictum* and its main vector *Culex quinquefasciatus* in the Hawaiian archipelago, which triggered a dramatic decline and extinction of the native Liwi honeycreeper (*Drepanis coccinea*)^14^. Similarly, the generalist parasites of the genus *Haemoproteus* caused mortality in several parrots maintained in captivity in Germany^15^. In addition, Haemosporidia parasites can impose strong selective pressures on their hosts^16–18^, and even cause a reduction in the longevity of their vectors^19^.

These deleterious effects on parasites on the population dynamics of their bird hosts may influence the mechanisms that drive diversity-disease patterns. These have been widely discussed in literature, especially because various ecological and epidemiological characteristics of host communities can affect the spread of pathogens^20,21^. For example, Ferraguti et al.^22^ found that host demographic factors affect the distribution and transmission of avian malaria. This may be because, by definition, parasites need hosts for food and habitat, so an increase in host diversity (richness) and abundance (or density) may initially increase the risk of infection^23^. However, if parasites infect more abundant and widely distributed hosts, or if the host is faced with a decision of prioritizing defense against parasites´ overgrowth, reproduction, and dispersal, then communities may be configured so that initially infected species are typically competent (acting as infection amplifiers), while later additions may be less frequent or act as diluting hosts^20,23,24^.

Most studies on the effect of host diversity on haemosporidian infections reported that host richness and not diversity influences this interaction^22,25,26^. Other studies have found that haemosporidian prevalence is positively associated with host density^27^ and host richness^28^, but negatively correlated with the density of non-competent host species, probably due to a dilution effect on disease transmission^25,26^. This suggests an important role of avian community structure and composition on parasite transmission^22,26,29^. Ecological models of the rate of per capita population change, such as Verhulst´s or Lotka-Volterra´s models, postulate a linear relationship of per capita growth with intra or interspecific density. However, for most organisms, the relationship between density and per capita growth rate is not well known and a linear relationship is only one possibility. Many of the relationships between density and the demographic components of population dynamics (e.g., survival, fecundity, reproductive age) are non-linear^30^, wich may also the case of parasite-host relationships. In the analysis of 205 biodiversity-disease relationships on 67 parasite species, Halliday et al.^23^ found that nonlinear hump-shaped relationships are common. However, in avian haemosporidian, host richness and diversity have been assessed only linearly without considering more complex polynomial functions that could increase model fitness.

Although some parasitic interactions have been characterized in detail^22,25,31^, little is known about the dynamics or even the patterns of parasites infections at the community level. Here, we conducted bird censuses and collected samples from wild birds in the areas surrounding three artificial dams to explore the role of the avian community on the infection numbers, prevalence and expected lineage richness of haemosporidian parasites. Since, according to the literature, the shape of the relationship depends on the dominance and richness of host species in the community, an amplification effect could be found in linear, polynomial, logarithmic, or exponential relationships favored by species susceptible to infection, whereas a dilution effect could be expected in communities with abundant non-competent (or less susceptible) bird species to infections.

## Results

### Host composition

The bird communities of the three dams showed a high level of diversity. Porce III presented the highest number of species (n=212) followed by Porce II (n=186) and Playas (n=158). However, we did not find any significant differences in richness (Chao), diversity (Shannon H') or dominance (D) among the bird communities across different areas or within each transect (Table S1, Supporting information; Kruskal-Wallis; *p*>0.05 in all cases).

### Molecular identification of avian blood parasites

We captured 863 birds and obtained blood samples from 678 individuals (90 species). Of these samples, 66 from 29 species were infected by blood parasites (prevalence 9.73%) (Table 1). The majority of infections (n=30) corresponded to *Haemoproteus* parasites, while 27 and 9 birds were infected by *Plasmodium* and *Leucocytozoon*, respectively. Sequencing of the positive samples revealed the circulation of 39 lineages in total (14 *Haemoproteus* lineages, 17 *Plasmodium* lineages and 8 *Leucocytozoon* lineages). Overall, thirteen of these lineages are new (6 *Haemoproteus* lineages infecting five bird species, 4 *Plasmodium* lineages infecting four species of birds and 3 *Leucocytozoon* lineages infecting three species of birds). There was no evidence of mixed infections by at least two parasite lineages. No significant differences were observed in the number of infections or in the prevalence of Haemosporidian parasites among the three dams or among different seasons (Kruskal-Wallis *p*>0.05). The complete list of parasite lineages identified is given in Table S2 (Supporting information).

**Table 1:** Number of infections and prevalence of Haemosporidian parasites found in birds in the study area.

Study area	Number of infections	Number of samples	Prevalence	*Plasmodium*		*Haemoproteus*		*Leucocytozoon*	
				Infec	Prev (%)	Infec	Prev (%)	Infec	Prev (%)
Playas	16	298	5.4%	3	1.01	8	2.68	5	1.68
Porce II	22	174	12.6%	11	6.32	9	5.17	2	1.15
Porce III	28	206	13.59%	13	6.31	13	6.31	2	0.97
Total	66	678	9.73%	27	3.98	30	4.42	9	1.33
p Kruskal–Wallis	0.87		0.34						

Infec: Number of infections, Prev: Prevalence.

The results regarding the prevalence of infection in each species, classified by parasite genus and sample site, are presented in Table S3 (Supporting information). Regarding total prevalence, information corresponding to 18 species is reported as they had more than four individuals sampled. At least 14 with a sample size higher than 4 individuals showed a zero prevalence of infections. The highest prevalence of infection was found for *Myarchus tuberculifer* (prevalence 40%, 2 positives out of 5 sampled), followed by *Arremon aurantiirostris* (37.5%, 9/24), *Catharus ustulatus* (36.3%, 8/22), *Saltator maximus* and *Catharus minumus* (prevalence 28.6%, 4/14 and 2/7, respectively). Interestingly, *A. aurantiirostris* exhibited a notably high prevalence of infection at Porce II and Porce III dams, 80% and 63%, respectively but none of the individuals captured in Playas were infected (0/11).

### Relationship between haemosporidian infections and bird communities

Table 2 shows the models that provide the most plausible explanation for the interaction between host community variables and the number of parasite infections (total and number of infections for *Plasmodium* and *Haemoproteus*), parasite prevalence (number of infections over the total number of samples evaluated at each site), and the estimated richness of *Haemoproteus* and *Plasmodium* lineages.

**Table 2:** Most plausible models according to the Akaike Information Criterion (AIC), for haemosporidian ~ bird interactions.

Model	Intercept	Chao	D	Chao^2^	Chao^3^	D^4^	log(D)	exp(D)	exp(Chao)	AICc	Delta	Weight	R^2^
Prevalence													
nullPrev	− 2.206	NA	NA	NA	NA	NA	NA	NA	NA	7.198	0.000	0.332	
Prevalence ~ Chao	− 2.199	0.000	NA	NA	NA	NA	NA	NA	NA	9.588	2.390	0.101	
Number of total infections													
Infections ~ D^4^	0.886	NA	NA	NA	NA	+	NA	NA	NA	99.154	0.000	0.550	0.470
nullInfec	1.012	NA	NA	NA	NA	NA	NA	NA	NA	103.055	3.901	0.078	
Number of infections by *Haemoproteus*													
nullInfec	0.223	NA	NA	NA	NA	NA	NA	NA	NA	75.029	0.000	0.245	
Infections ~ exp(Chao)	0.192	NA	NA	NA	NA	NA	NA	NA	0.000	77.134	2.106	0.086	
Number of infections by *Plasmodium*													
Infections ~ exp(D)	− 5.118	NA	NA	NA	NA	NA	NA	2.290	NA	74.925	0.000	0.212	0.160
Infections ~ D	− 4.005	NA	5.006	NA	NA	NA	NA	NA	NA	74.927	0.002	0.212	0.160
Infections ~ log(D)	0.875	NA	NA	NA	NA	NA	3.798	NA	NA	74.938	0.013	0.211	0.160
nullInfec	0.118	NA	NA	NA	NA	NA	NA	NA	NA	76.714	1.789	0.087	
Infections ~ Chao	− 0.577	0.051	NA	NA	NA	NA	NA	NA	NA	77.156	2.232	0.070	
Relative importance			0.330				0.330	0.330					
Richness* Haemoproteus* lineages													
Chao Haem ~ Chao^2^	− 0.004	NA	NA	+	NA	NA	NA	NA	NA	67.992	0.000	0.426	0.310
Chao Haem ~ Chao^3^	− 0.007	NA	NA	NA	+	NA	NA	NA	NA	70.651	2.659	0.113	
Richness *Plasmodium* lineages													
Chao Plas ~ D	− 4.005	NA	5.006	NA	NA	NA	NA	NA	NA	74.927	0.000	0.575	0.160
nullInfec	0.118	NA	NA	NA	NA	NA	NA	NA	NA	76.714	1.787	0.235	
Chao Plas ~ Chao	− 0.577	0.051	NA	NA	NA	NA	NA	NA	NA	77.156	2.230	0.189	

**Δ**(AICc) = [AICc—minAICc]; **ω**(AICc) = the rounded second order Akaike weights. Chao = Bird richness, D = Bird dominance.

Specifically, regarding the number of infections, the best model includes a fourth-order polynomial relationship with host dominance. However, when examining the number of infections for each genus separately, the best model for *Plasmodium* infections includes the exponent of host dominance. In addition, three other models (ΔAICc < 2), including the null model, were considered equally plausible. These alternative models propose dominance in linear, exponential, and logarithmic relationships (Table 2). On the other hand, the number of *Haemoproteus* infections and prevalence were best explained by the null model, suggesting that these factors may not be influenced by bird community dominance or richness.

The relationship between estimated *Haemoproteus* richness and estimated *Plasmodium* richness was best explained by models incorporating second-order polynomial richness and linear dominance relationships, respectively. However, the ΔAICc value for *Plasmodium* richness models suggests that the null model might also be equally plausible.

**Figure 1:**
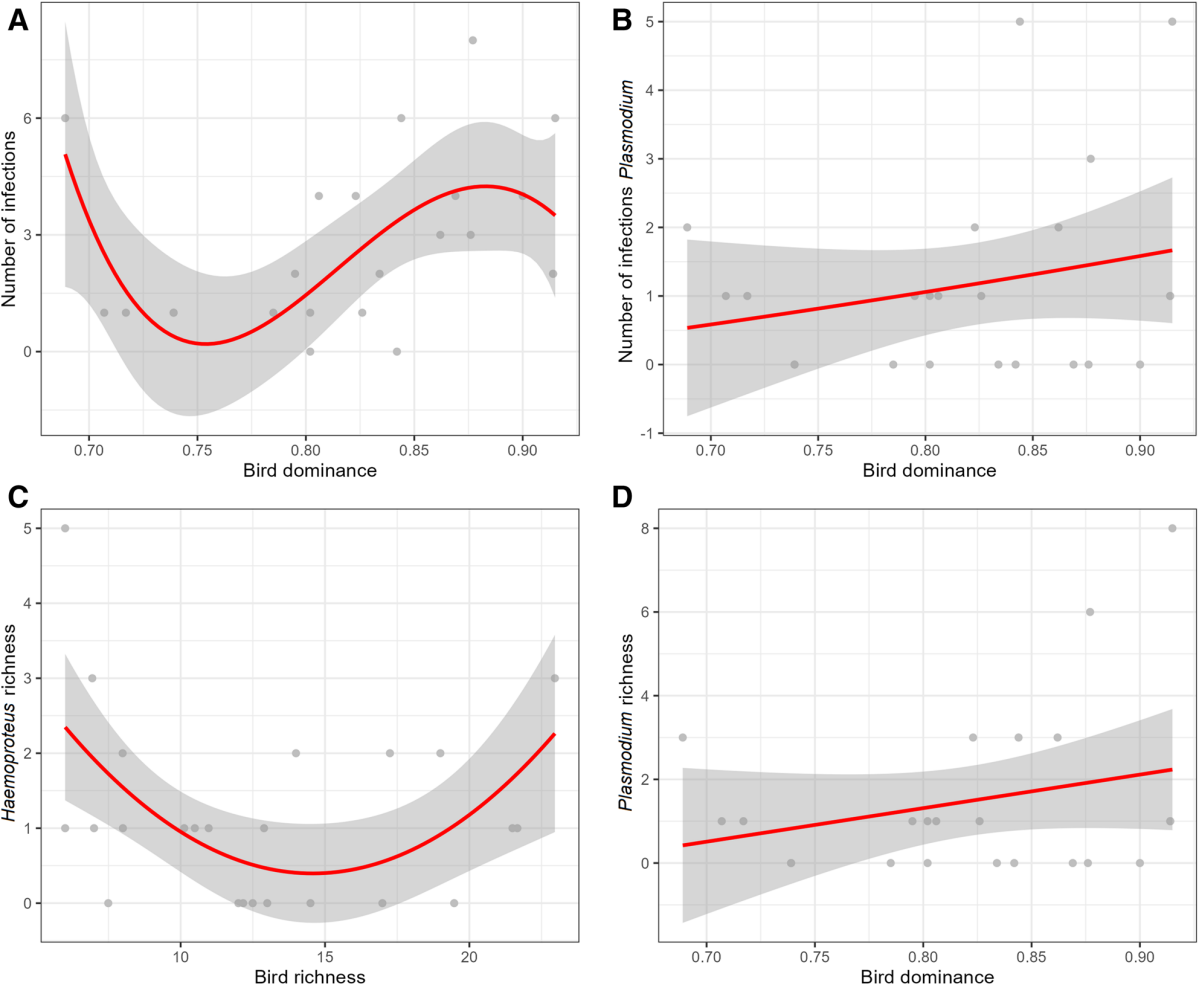
Relationship between (**A**). Number of total infections (Infections ~ D^4^), (**B**). Number of infections by *Plasmodium* (Infections ~ exp(D)), (**C**). Expected richness of *Haemoproteus* lineages (Chao Haem ~ Chao^2^) and (**D**). Expected richness of *Plasmodium* lineages (Chao Plas ~ D) with the dominance and richness of the bird community**.** The grey area corresponds to the standard error.

In summary, models explaining the relationship between the number of infections (excluding the number of infections by *Haemoproteus*) and expected *Plasmodium* richness indicate that host community dominance is directly and positively related to linear, logarithmic, and exponential growth, suggesting an amplification effect (Fig. 1). However, second and fourth-order relationships suggest that this effect is present within specific ranges of dominance and richness. This effect may be driven by the abundance of susceptible species for infection, such as *Manacus manacus*, *Machaeropterus striolatus*, *M. tuberculifer*, and *A. aurantiirostris*, which showed the highest number of infections in certain evaluated transects (Figure S1, Supporting information).

## Discussion

In this study, we examined the influence of the avian community on the infection numbers, prevalence and expected lineage richness of haemosporidian parasites. Additionally, we investigated the relationship these factors by considering not only linear patterns but also exploring more complex polynomial functions. Our findings revealed a significant amplification effect in positive linear, exponential, logarithmic, and polynomial relationships, influenced by host abundance.

We found a high and similar bird richness in the three study areas evaluated. These findings are in agreement with research carried out in the Neotropics, where there is a great heterogeneity of habitats and climates, even in small patches of vegetation a high richness of species is found^32^.

The number of infections (66) and the prevalence (9.73%) were similar to those reported for the Neotropics. More explicitly, in areas near to these three dams, Martínez-Alvarado^33^ found a prevalence of 14.8% and Pulgarín et al.^34^ reported a prevalence of 14.3%. However, clear differences were found with respect to the parasite genera studied. *Haemoproteus* and *Plasmodium* presented a similar number of infections, 30 and 27, which represented a prevalence of 4.42% and 3.98%, respectively. The literature reports that both genera present a heterogeneous distribution, a highly variable prevalence (0–100%), and a great diversity of lineages^35,36^, a pattern also found here. In an ecologically similar area, Anjos et al.^37^ reported a comparable prevalence of *Plasmodium* (2.9%, 13 positive samples out of 445 birds) to the findings of our study, which supports of a low prevalence of this genus in ecosystems surrounding dams.

On the other hand, we found that the number of infections of *Leucocytozoon* was low (n=9, prevalence 1.33%) with 5 out of the 9 *Leucocytozoon* infections found in migratory species including *Catharus minimus* (n=1) and *Catharus ustulatus* (n=4). *Leucocytozoon* lineages have been detected in resident bird species of Neotropical lowlands^38^. Lotta-Arevalo^35^ and Matta and Rodriguez^36^ found that the prevalence of *Leucocytozoon* ranged from 10% to 25% in lowlands in general, in migratory birds. The low prevalence of *Leucocytozoon* could be attributed to the limited availability of susceptible vectors for transmission and maintenance of the parasite cycle. However, the existence of reports in territorial species suggests the presence of competent vectors maintaining the circulation of parasites from either migratory birds or resident chronically infected hosts in such places. Further studies should evaluate the prevalence of *Leucocytozoon* using both molecular and microscopic analysis in birds in the area, as well as to identify the potential vectors involved in its transmission.

In this study we found no differences in the number of infections and prevalence between seasons (dry and wet), this finding agrees with Lopes et al.^39^ who found that hosts, parasites, and their interactions do not vary under temperature and precipitation oscillations, being stable throughout the seasons. However, when evaluated within the community, although the number of infections and prevalence between sites is similar, this pattern is not repeated between species. For example, in the case of *A. aurantiirostris*, high prevalences were found in Porce II and Porce III (80% and 62.5%, respectively). Species of this family (Passerelidae, formerly Emberizidae) usually show high prevalence of infection (>40%) in the Neotropics^40,41^. However, our results suggest that local differences may exist in the prevalence of infection, as none of the 11 *A. aurantiirostris* individuals sampled in Playas were infected. These differences could be due to different factors. Firstly, it is possible that the availability of food, associated with greater vegetation cover, favors the response of the species to infection, where better resources improve the body condition of birds, making individuals less susceptible to infection^42^. In support of this hypothesis the Playas dam, has a high plant diversity^43^ that could potentially explain the absence of parasitic infection in *A. aurantiirostris* at this locality. Several studies indicate that the availability of food for birds increases the innate and adaptive response to immune challenges produced by emerging diseases^44^. In addition, vector ecology may affect the infection patterns, as insect vectors may be favored by open areas showing greater diversity in areas with heterogeneous anthropized cover, including agricultural landscapes^45^ and pastures^46^. The vector community is an important predictor, for example, in explaining variation in *Plasmodium* prevalence in birds^22^. Future efforts should implement the characterization of vectors of avian haemosporidian in the Neotropics.

We observed significant relationships between the number of infections, estimated *Haemoproteus* and *Plasmodium* richness, and bird dominance and richness. However, we found no significant relationship between haemosporidian prevalence and the number of *Haemoproteus* infections and the community variables considered.

The relationships between the polynomial, exponential, logarithmic and linear terms of host dominance and parasite variables were not unexpected due to the high number of species recorded (270) and the possibility that a high host diversity may provide different niches for different parasite lineages^41,47^, favoring an amplification pattern. This could be due to the availability of host-saturated environments under favorable environmental conditions for the development of the parasite life cycle^47^. Species vary in their diluting and amplifying capacity depending on their abundance, susceptibility, and transmission potential, so that certain species may disproportionately affect disease risk^20,31,48,49^. This makes necessary not only to evaluate the patterns associated with the diversity of a host community but also to analyze independently whether each host is competent or not for the parasite and whether their abundances generate a dilution or amplification effect in the community, as has already been evaluated with *Passer domesticus* in Spain^50^. In the second and fourth-order polynomial relationships, we have observed both amplification and dilution patterns occurring simultaneously within the same system. Although previous studies have shown that the dilution effect can be scale-dependent^23^, we demonstrated that dilution and amplification could co-occur and the resultant overall effect will be determined by which of the two effects is stronger^49^ and by the identity and abundance of the species involved in each community.

In the community studied, species such as *M. manacus*, *M. striolatus*, *M. tuberculifer* and *A. aurantiirostris*, which had the highest number of infections in almost all the sites sampled, these species may generate the amplification effect in these sites (Figure S1, Supporting information). Huspeni and Lafferty^51^ and Ferreira Junior et al.^46^ found that, host species present higher prevalences and richness of parasites during restoration or succession processes, than in more conserved sites. In addition, these bird species are usually found in poorly conserved areas and under restoration processes^52^. However, as new non-competent (diluting) species become available and forests become older and the species assemblage stabilizes, one would expect that the abundance of competent (amplifying) species will be reduced and, therefore, exposure to the parasite will also decrease^20^.

Although some parasite interactions have been characterized in detail^22,25,27^, little is known about the dynamics and patterns of parasites at the community level. For example, Ricklefs et al.^53^, evaluated the relationships between hosts and prevalence of malaria parasites, finding that the latter presented a U-shaped relationship (quadratic regression) with host sample size. However, they assumed abundances of individuals captured in mist-nets as a relative proportion of abundances in communities. In this study, we used census-reported abundances to estimate the dominance index, which may represent a good proxy for community-level abundances. The scarce representation of studies at the avian community and haemosporidian infection level that include census data within the analysis represents a novelty in the characterization of these interactions. However, it is essential to further evaluate the different actors involved in these interactions, such as mosquitoes and other vertebrate groups, which although not considered within these analyses, could be an important factor in explaining the patterns of infections in birds.

In conclusion, our results provide valuable information on the diversity of blood parasites infecting birds in a hyperdiverse and poorly studied area. In addition, we provide evidence for the role of host community as a relevant factor determining the parasite infections in wild birds supporting the complex relationships between these components.

## Methods

### Study area

**Figure 2:**
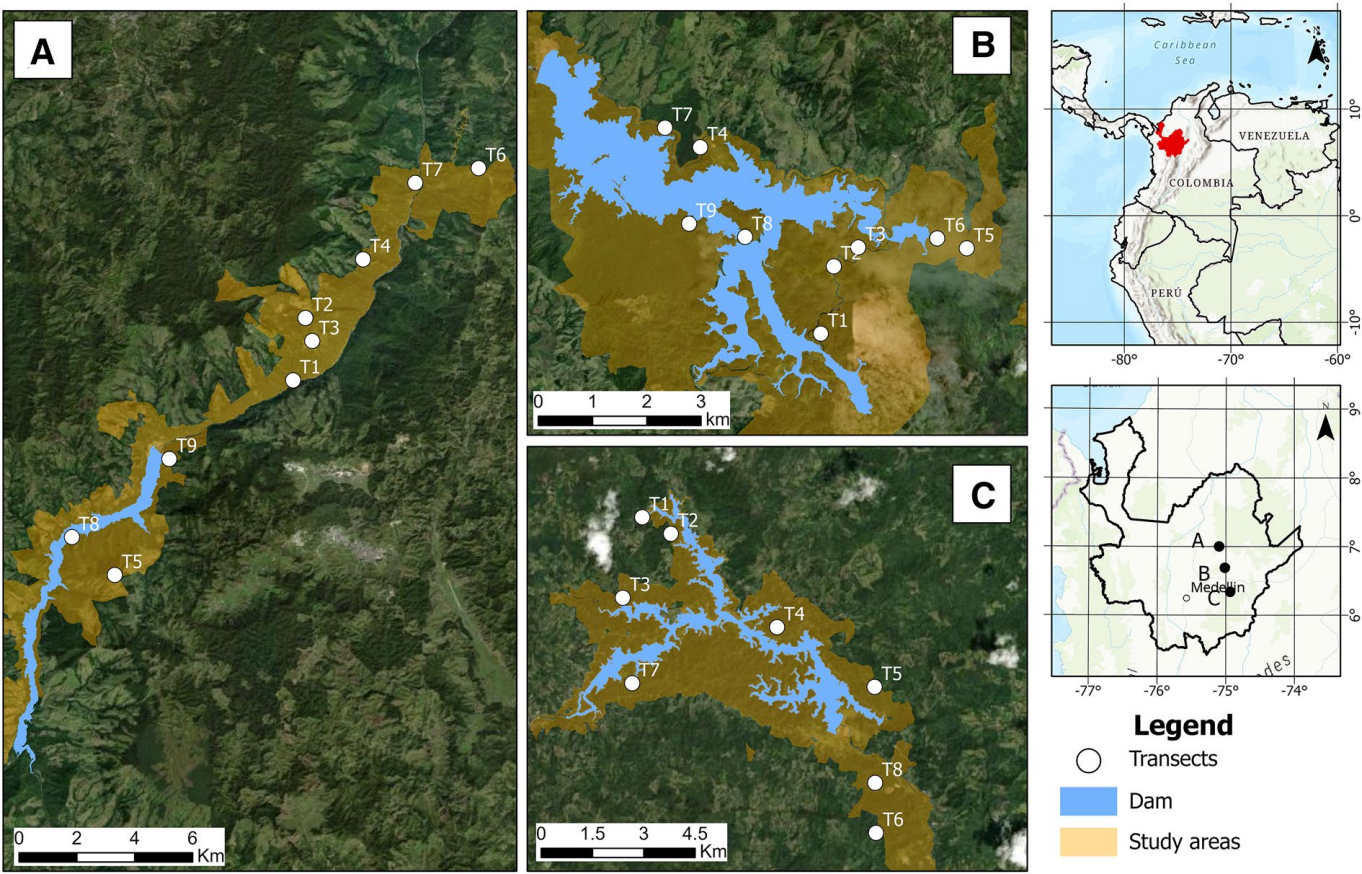
Study areas in the department of Antioquia-Colombia (North of South America) including Porce III (**A**), Porce II (**B**) and Playas (**C**).

This study was conducted in three forested areas around artificial dams in Antioquia, Colombia (Fig. 2). These areas are located in the tropical rainforest life zone, with a mean annual temperature of 22.1°C (range = 13.9–33.4 °C). The average annual relative humidity is 83.3% and annual precipitation ranges between 2300 and 3300 mm^54^. The studied dams have been under restoration actions for around 40 years, hence, vegetation covers comprises secondary forests with an advanced successional stage, and high natural dynamics and complex conditions in the structure and floristic composition. Some of these forests have well-defined vegetation strata, with a predominance of medium height canopy (20 m), with emergent trees that usually reach the maximum diameter at breast height values^43^.

We sampled Playas between February–June 2018 and Porce II and Porce III between March–October 2019, covering one dry and one wet season at each site. To sample bird communities structure and composition, we registered birds at point counts and transects of different length, through auditive and visual data^55,56^. We established transects from 600 meters to 1 km, where we placed three-to-four-point counts at a distance of at least 300 m among each other (Table S4, Supporting information). These point counts were used to record all the birds observed or heard within a radius of 100 m, for 15 min. Visual records of species were conducted using Nikon Monarch 8×42 binoculars and auditive by recording with a Marantz recorder with Sennheizer omnidirectional microphones, the later being used to clarify taxonomic aspects of identifications. Simultaneously, we placed ten to twelve mist-nest at sites close to vegetation plots taking the mean coordinate between mist-nets sets on each transect, ensuring spatial independence between sampled areas. Mist-nets remained open between 6:00 h and 14:00 h for two days at each site, being checked at intervals of 15 min or less, depending on weather and bird activity. A trained person was in charge of removing the captured birds and taking morphological measurements as well as blood samples. Birds were blood sampled (~20 µl) by brachial venipuncture. Blood samples were stored in Queen lysis buffer and kept frozen in the laboratory until further molecular analyses^57^. At the end of the procedure and before releasing, each individual bird was marked with a cut in the most distal part of a tail feather to prevent resampling.

### Molecular diagnosis of haemosporidians

We extracted DNA from blood samples following the salting-out method^58^. DNA quality was checked by amplification of a fragment of the avian mitochondrial ND4 gene with ND4 and LEU primers^59^. The amplified DNA was observed through 2% agarose gel electrophoresis stained with gel red (®biotium). Samples with good DNA quality (those that successfully amplified the avian ND4 fragment) were used for the molecular diagnosis of *Plasmodium*, *Haemoproteus* and *Leucocytozoon* infections. These parasites were screened using a nested PCR protocol to amplify a fragment of the mitochondrial gene cyt-b of the parasite (Table S5, Supporting information). Briefly, a universal PCR was performed to amplify a fragment common to all three parasites in the first PCR reaction. Subsequently, a nested PCR was performed to separately amplify parasites of the genera *Plasmodium/Haemoproteus*^60^ and *Leucocytozoon*^61^. Each PCR was performed at least twice to avoid the occurrence of false negatives. The reactions included a positive control (confirmed sample with positive infection by sequencing and microscopy) and a negative control (reaction without DNA). Amplification products were visualized in 2% agarose gels. Amplicons were purified using Exonuclease I (20 u/µl) and Calf intestine alkaline phosphatase (CIP 10 u/µl) according to the manufacturer's instructions and sequenced by Macrogen Inc (Korea). The forward and reverse sequences were aligned and edited in Geneious version 2020.2.4^62^ and then compared with those available in GenBank and Malavi databases^63^. New lineages were confirmed by sequencing at least twice both forward and reverse directions and deposited in GenBank (Table S2, Supporting information).

### Statistical analysis

We calculated the alpha diversity index of the bird community from the censuses conducted at the point counts and transects, taking a sub-sample of the records of species captured with mist-nets, since these are the effective species for which we were able to determine their infection status. Therefore, including all recorded species may bias the results because the infection status of many species is unknown. Both the Shannon-Wiener index (H'; hereafter diversity) and the Simpson’s reciprocal index (D; hereafter dominance) were calculated. Diversity (H') incorporates the number of species in a community and their relative abundances. This index presents a value considering the species richness and the evenness of each community, with higher values for richer communities (>3) and greater equity between species^64^. Dominance (D) is the probability of an intraspecific encounter, in other words, the probability that, if two individuals are taken randomly from the community, both are of the same species. This index varies from 0 to 1, being higher for communities with one or several species with high dominance^65^. Finally, the estimated host species richness was calculated with the Chao estimator^50^ which considers the frequency of each species detected and calculates the estimated value of richness per site. We used the non-parametric Kruskal–Wallis test to identify differences in the estimated indices among the evaluated sites.

To explore the effects of host composition (explanatory variable) on the diversity, richness and prevalence of haemosporidia (response variable), the total number of infections and the estimated richness of lineages were calculated with the Chao estimator^50^. This estimator considers the frequency of each lineage detected and calculates the estimated value of the richness by parasite genus. The prevalence of haemosporidia (proportion of infected host individuals) was calculated as the number of infected individuals over the total number of blood-sampled individuals at each site. *Leucocytozoon* was excluded from further analyses due to the low prevalence found in the area. We found no significant differences in the number of infections between transects, dams and seasons, so analyses were performed for the entire data set.

Finally, we evaluated the relationships between the number of infections, prevalence, and estimated richness (Chao) of *Haemoproteus* and *Plasmodium* with the estimated richness (Chao) and dominance (D) of the bird communities at each site. Diversity (H') was excluded from the analysis as it was highly correlated with richness (r=0.85) and dominance (r=0.93). For this, we ran generalized linear models (GLM) evaluating the degree of fitness in linear, logarithmic, exponential and polynomial models of the explanatory variables of dominance and richness (Chao). The normal distribution of all predictors and model residuals was checked by using qqplots in R software. We used a binomial distribution for the prevalence models, while the number of infections and the richness of the haemosporidian lineages were fitted using a Poisson distribution. A different set of models was built for each of the response variables: number of infections (total and number of infections by *Plasmodium* and *Haemoproteus*), prevalence, estimated richness of *Haemoproteus* and *Plasmodium* lineages, with all the possible combinations of the predictors (Chao estimated richness and dominance of the bird community). To select the best model, we used the Akaike Information Criterion (AIC). This procedure penalizes the models according to the number of data and parameters included^66^. The AIC selection criteria is based on an estimation of the Kulback-Leiber inequality that proposes a mechanism to measure a distance between two functions, where the best model will be the one with the lowest AIC. The models with a ΔAICc ≤ 2 were selected. Additionally, the models were assessed for their goodness of fit using pseudo R^2^. Statistical analyses were conducted in R using packages vegan, stats, pscl and MuMInc^67^.

### Ethical approval

All experiments and protocols were approved for the committee of ethics of the Universidad de Antioquia and the of resolution 0524 of ANLA (National Environmental Licensing Authority). The study was performed under proper legislation of the Colombian law and following the Code of Ethics of the Animal Behavior Society, and the ABS/ASAB Guidelines for the use of animals in research and teaching. Only blood samples were collected, and all birds were returned to their natural environment. Animals were not kept in captivity and were not exposed to any experimental treatment. The capture/collection of biological samples and specimens is covered by a permit for the collection of wild specimens for non-commercial purposes, issued by the National Environmental Licensing Authority (ANLA) through resolution 0524 to the Universidad de Antioquia, which includes the Grupo de Ecología y Evolución de Vertebrados. Moreover, the export of biodiversity samples in Colombia for genetic (phylogenetic) analysis, is regulated by the ANLA, which grants an export permit for Scientific Research purposes. Since Universidad de Antioquia holds a collection permit, all research activities with wild specimens or biological samples are performed under Colombian law. All the authors complied with the ARRIVE guidelines and the submission guidelines for manuscript.

## Supplementary Information

Supplementary Information.

## Data Availability

The datasets used and/or analysed during the current study available from the corresponding author on reasonable request.
