# Supplementary material for: Bird community effects on avian malaria infections
Source: Sci Rep. 2023 Jul 19;13:11681. doi: 10.1038/s41598-023-38660-2 (PMC10356947; doi:10.1038/s41598-023-38660-2)
Supplement: Supplementary file 1 — Supplementary Information. [file 41598_2023_38660_MOESM1_ESM.pdf]

Bird community effects on avian malaria infections

Juliana Tamayo-Quintero[1*], Josué Martínez de la Puente[2], Miriam San-José[3], Catalina González-Quevedo[1] and Héctor F. Rivera-Gutierrez[1]

[1] Grupo de Ecología y Evolución de Vertebrados, Universidad de Antioquia, Colombia
[2] Departamento de Parasitología, Facultad de Farmacia, Universidad de Granada, España
[3] Charles Darwin Research Station, Charles Darwin Foundation, Ecuador
[*] juliana.tamayoq@udea.edu.co

Supplementary Table 1. Diversity index for each evaluated transect. In the Bird diversity (Mist-nets) column only records of species caught in mist nets were considered. Chao= Bird richness, D= Bird dominance, H´= Shannon index, *Haem*= *Haemoproteus*, *Plas*= *Plasmodium*, *Leu*= *Leucocytozoon*.

Dam	Site	Richness of lineages			Number of infections			Total number of infections	Number of samples	Prevalence	Bird diversity (Census)			Bird diversity (Mist-nets)	
		Haem	*Plasm*	*Leuc*	*Haem*	*Plasm*	*Leuc*				Chao	H´	D	Chao	D
Playas	T1	3	0	1	2	0	1	3	36	0.083	103.188	3.822	0.967	22.962	0.876
Playas	T2	2	0	1	3	0	1	4	54	0.074	102.563	3.895	0.971	17.249	0.900
Playas	T3	2	0	1	3	0	1	4	37	0.108	108.178	3.799	0.969	13.995	0.869
Playas	T4	0	0	3	0	0	2	2	40	0.050	75.333	3.614	0.958	14.497	0.834
Playas	T5	0	1	0	0	1	0	1	29	0.034	92.045	3.633	0.960	19.474	0.826
Playas	T6	0	1	0	0	1	0	1	49	0.020	83.844	3.531	0.947	16.988	0.717
Playas	T7	0	0	0	0	0	0	0	31	0.000	92.225	3.834	0.971	12.000	0.842
Playas	T8	0	1	0	0	1	0	1	19	0.053	114.333	3.840	0.966	12.496	0.802
Porce II	T9	0	0	0	0	0	0	0	19	0.000	107.014	4.036	0.976	12.165	0.802
Porce II	T10	0	1	0	0	1	0	1	20	0.050	120.012	4.051	0.976	7.493	0.707
Porce II	T11	1	0	0	1	0	0	1	23	0.043	90.383	3.848	0.966	7.000	0.785
Porce II	T12	2	1	0	3	1	0	4	20	0.200	111.742	4.002	0.973	7.990	0.806
Porce II	T13	5	3	0	4	2	0	6	42	0.143	97.280	3.864	0.965	6.000	0.689
Porce II	T14	0	3	1	0	5	1	6	31	0.194	91.000	3.787	0.964	13.000	0.844
Porce II	T15	1	3	1	1	2	1	4	21	0.190	92.345	3.802	0.961	8.000	0.823
Porce II	T16	0	0	0	0	0	0	0	1	0.000	67.846	3.164	0.911	0.000	0.000
Porce II	T17	0	0	0	0	0	0	0	0	-	84.941	3.643	0.961	0.000	0.000
Porce III	T18	2	6	1	4	3	1	8	34	0.235	102.673	3.889	0.967	18.996	0.877
Porce III	T19	1	0	0	1	0	0	1	8	0.125	105.000	3.896	0.967	10.971	0.739
Porce III	T20	1	8	0	1	5	0	6	32	0.188	132.774	4.117	0.976	21.493	0.915
Porce III	T21	1	1	0	1	1	0	2	32	0.063	128.273	4.194	0.979	21.663	0.914
Porce III	T22	3	0	1	2	0	1	3	8	0.375	78.250	3.272	0.937	6.939	0.377
Porce III	T23	1	1	0	1	1	0	2	20	0.100	78.407	3.287	0.932	12.889	0.914
Porce III	T24	1	1	0	1	1	0	2	30	0.067	117.853	4.124	0.978	10.123	0.795
Porce III	T25	1	3	0	1	2	0	3	27	0.111	130.857	3.880	0.969	10.491	0.862
Porce III	T26	1	0	0	1	0	0	1	15	0.067	87.000	3.669	0.967	6.000	0.739

In order to sample the structure and composition of bird communities, we registered birds with point counts and different length transects (Site in the table). We established transects from 600 meters to 1 kilometer, where we placed three-to-four-point counts at a distance of at least 300 meters each. Based on data recorded in these censuses, we calculated the index of alpha diversity of the bird community taking a sub-sample from the records of species caught in mist-nets, since these are the effective species for which we were able to determine their infection status.

Supplementary Table 2. Lineages of Haemosporidia record in the study area.

Transect	Dam	Code	Host species	Genus	Lineage MalAvi	Accesion-Genbank
PT1	Playas	1	*Machaeropterus striolatus*	*Haemoproteus*	MACSTR01	New
PT1	Playas	23	*Machaeropterus striolatus*	*Leucocytozoon*	POEHUD01	
PT1	Playas	48	*Manacus manacus*	*Haemoproteus*	MANMAN01	
PT2	Playas	58	*Saltator maximus*	*Haemoproteus*	LEPCOR03	
PT2	Playas	87	*Manacus manacus*	*Haemoproteus*	MANMAN01	
PT2	Playas	89	*Manacus manacus*	*Haemoproteus*	MANMAN01	
PT3	Playas	110	*Catharus ustulatus*	*Leucocytozoon*	CATMIN01	
PT3	Playas	126	*Machaeropterus striolatus*	*Haemoproteus*	MACSTR126	New
PT3	Playas	134	*Catharus minimus*	*Haemoproteus*	MANMAN01	
PT4	Playas	184	*Catharus ustulatus*	*Leucocytozoon*	HYLMUS02	
PT4	Playas	203	*Catharus ustulatus*	*Leucocytozoon*	CATUST203	New
PT2	Playas	211	*Manacus manacus*	*Haemoproteus*	MANMAN01	
PT2	Playas	222	*Catharus minimus*	*Leucocytozoon*	CATMIN222	New
PT6	Playas	259	*Machaeropterus striolatus*	*Plasmodium*	FORANA02	
PT6	Playas	275	*Machaeropterus striolatus*	*Plasmodium*	MACSTR275	New
PT8	Playas	333	*Formicarius analis*	*Plasmodium*	FORANA02	
PIIIT1	Porce 3	414	*Catharus ustulatus*	*Plasmodium*	CATUST21	
PIIIT1	Porce 3	416	*Manacus manacus*	*Haemoproteus*	LEPCOR03	
PIIIT1	Porce 3	424	*Manacus manacus*	*Haemoproteus*	LEPCOR03	
PIIIT1	Porce 3	432	*Parkesia noveboracensis*	*Leucocytozoon*	SETCOR17	
PIIIT2	Porce 3	444	*Arremon aurantiirostris*	*Haemoproteus*	ARRAUR03	
PIIIT3	Porce 3	473	*Saltator maximus*	*Plasmodium*	PADOM11	
PIIIT3	Porce 3	476	*Lepidocolaptes souleyetii*	*Plasmodium*	PADOM09	
PIIIT3	Porce 3	477	*Thamnophilus atrinucha*	*Plasmodium*	WILPOE17	
PIIIT4	Porce 3	490	*Habia gutturalis*	*Plasmodium*	EMBHER01	
PIIIT4	Porce 3	497	*Chlorophanes spiza*	*Haemoproteus*	CHLSPI497	New
PIIT2	Porce 2	539	*Myiarchus tuberculifer*	*Plasmodium*	MYRLON02	
PIIT3	Porce 2	550	*Manacus manacus*	*Haemoproteus*	LEPCOR03	
PIIIT3	Porce 3	577	*Arremon aurantiirostris*	*Plasmodium*	TACRUB04	
PIIIT3	Porce 3	579	*Arremon aurantiirostris*	*Plasmodium*	TACRUB04	
PIIIT3	Porce 3	581	*Arremon aurantiirostris*	*Haemoproteus*	ARRAUR03	
PIIIT1	Porce 3	606	*Cercomacroides tyrannina*	*Plasmodium*	CERTYR606	New
PIIIT1	Porce 3	607	*Arremon aurantiirostris*	*Haemoproteus*	ARRAUR03	
PIIT1	Porce 3	609	*Henicorhina leucosticta*	*Haemoproteus*	LEPCOR03	
PIIT1	Porce 3	609	*Henicorhina leucosticta*	*Plasmodium*	EMBHER01	
PIIT4	Porce 2	647	*Arremon aurantiirostris*	*Plasmodium*	BAEBIC02	
PIIT4	Porce 2	653	*Islerothraupis luctuosa*	*Haemoproteus*	TACCRI01	
PIIT4	Porce 2	657	*Arremon aurantiirostris*	*Haemoproteus*	ARRAUR03	
PIIT4	Porce 2	658	*Arremon aurantiirostris*	*Haemoproteus*	ARRAUR03	
PIIIT6	Porce 3	666	*Tangara gyrola*	*Haemoproteus*	TANCHI01	
PIIIT6	Porce 3	666	*Tangara gyrola*	*Leucocytozoon*	TANGYR666	
PIIIT6	Porce 3	667	*Stilpnia larvata*	*Haemoproteus*	STILAR667	New
PIIIT6	Porce 3	669	*Stilpnia larvata*	*Haemoproteus*	TANCYA02	
PIIIT7	Porce 3	681	*Tachyphonus delatrii*	*Haemoproteus*	HYPRO01	
PIIIT9	Porce 3	714	*Gymnopithys bicolor*	*Haemoproteus*	GYMBIC714	New
PIIT5	Porce 3	721	*Manacus manacus*	*Haemoproteus*	LEPCOR03	
PIIT5	Porce 2	726	*Manacus manacus*	*Haemoproteus*	LEPCOR03	
PIIT5	Porce 2	728	*Arremon aurantiirostris*	*Haemoproteus*	ARRAUR03	
PIIT5	Porce 2	738	*Manacus manacus*	*Haemoproteus*	MANMAN738	New
PIIT5	Porce 2	744	*Ceratopipra erythrocephala*	*Haemoproteus*	LEPCOR03	
PIIT6	Porce 2	745	*Myiarchus tuberculifer*	*Plasmodium*	MYRLON02	

Transect	Dam	Code	Host species	Genus	Lineage MalAvi	Accesion-Genbank
PIIT5	Porce 2	757	*Sporophila nigricollis*	*Plasmodium*	TACRUB04	
PIIT6	Porce 2	760	*Tangara inornata*	*Plasmodium*	RAMCAR01	
PIIIT6	Porce 3	796	*Mionectes olivaceus*	*Plasmodium*	MIOOLE796	New
PIIIT8	Porce 3	799	*Geothlypis philadelphia*	*Plasmodium*	BAEBIC02	
PIIIT8	Porce 3	800	*Mionectes oleagineus*	*Plasmodium*	MIOOLE800	New
PIIT7	Porce 3	824	*Basileuterus rufifrons*	*Plasmodium*	PADOM09	
PIIT7	Porce 2	827	*Catharus ustulatus*	*Plasmodium*	MELMEL01	
PIIT7	Porce 2	836	*Catharus ustulatus*	*Haemoproteus*	CATUST22	
PIIT7	Porce 2	840	*Catharus ustulatus*	*Leucocytozoon*	CATMIN01	
PIIT7	Porce 2	840	*Catharus ustulatus*	*Plasmodium*	CATUST21	
PIIT6	Porce 2	864	*Vireo olivaceus*	*Plasmodium*	MELME02	
PIIT6	Porce 2	864	*Vireo olivaceus*	*Leucocytozoon*	VIROLI864	New
PIIT6	Porce 2	865	*Saltator maximus*	*Plasmodium*	PADOM11	
PIIT6	Porce 2	868	*Saltator maximus*	*Plasmodium*	PADOM11	
PIIT6	Porce 2	873	*Ramphocelus dimidiatus*	*Plasmodium*	RAMCAR01	

The table presents all positive samples (66) of the species captured and analyzed in mist-nets (678), including the lineage name according to the Malavi databases[45]. New lineages were confirmed by sequencing at least twice both forward and reverse.

Supplementary Table 3. Species with the highest number of samples evaluated (≥4), in order of prevalence (Prev) in the study area. The prevalence corresponds to the total number of infections over the total number of samples (N).

Species	Playas					Porce II					Porce III					Total				
	**N**	**Plas**	**Haem**	**Leu**	**Prev**	**N**	**Plas**	**Haem**	**Leu**	**Prev**	**N**	**Plas**	**Haem**	**Leu**	**Prev**	**N**	**Plas**	**Haem**	**Leu**	**Prev**
Myiarchus tuberculifer	2	0	0	0	0	3	2	0	0	-	0	0	0	0	0	5	2	0	0	0.4
Arremon aurantiirostris	11	0	0	0	0	5	1	3	0	0.8	8	2	3	0	0.63	24	3	6	0	0.38
Catharus ustulatus	10	0	0	3	0.3	6	2	1	1	0.67	6	1	0	0	0.17	22	3	1	4	0.36
Catharus minimus	5	0	1	1	0.4	2	0	0	0	-	0	0	0	0	-	7	0	1	1	0.29
Saltator maximus	6	0	1	0	0.17	4	2	0	0	0.5	4	1	0	0	0.25	14	3	1	0	0.29
Thamnophilus atrinucha	1	0	0	0	0	0	0	0	0	0	3	1	0	0	0	4	1	0	0	0.25
Henicorhina leucosticta	3	0	0	0	-	1	0	0	0	-	5	1	1	0	0.4	9	1	1	0	0.22
Cercomacroides tyrannina	1	0	0	0	-	0	0	0	0	-	4	1	0	0	0.25	5	1	0	0	0.2
Sporophila nigricollis	0	0	0	0	0	3	1	0	0	-	3	0	0	0	0	6	1	0	0	0.17
Tachyphonus delatrii	0	0	0	0	-	0	0	0	0	-	9	0	1	0	0.11	9	0	1	0	0.11
Ceratopipra erythrocephala	0	0	0	0	-	8	0	1	0	0.13	2	0	0	0	-	10	0	1	0	0.1
Manacus manacus	51	0	4	0	0.08	33	0	3	0	0.09	24	0	3	0	0.13	108	0	10	0	0.09
Gymnopithys bicolor	0	0	0	0	0	9	0	0	0	0	2	0	1	0	-	11	0	1	0	0.09
Ramphocelus dimidiatus	3	0	0	0	0	3	1	0	0	-	6	0	0	0	0	12	1	0	0	0.08
Basileuterus rufifrons	2	0	0	0	-	5	0	0	0	0	6	1	0	0	0.17	13	1	0	0	0.08
Machaeropterus striolatus	68	2	2	1	0.07	21	0	0	0	0	13	0	0	0	0	102	2	2	1	0.05
Habia gutturalis	15	0	0	0	0	4	0	0	0	0	4	1	0	0	0.25	23	1	0	0	0.04
Mionectes oleagineus	28	0	0	0	0	25	0	0	0	0	29	1	0	0	0.03	82	1	0	0	0.01

Results of the prevalence of infection in each species with respect to the parasite genera and sample site are shown in this table. Regarding the total prevalence, of the 29 species that presented infections, only 18 of them were considered in the table because they had more than four individuals sampled. However, it is important to mention that at least 14 other species met this criterion but did not present infections (zero prevalent species). The prevalence of haemosporidian (proportion of infected host individuals) was calculated as the number of infected individuals over the total number of blood sampled individuals at each species.

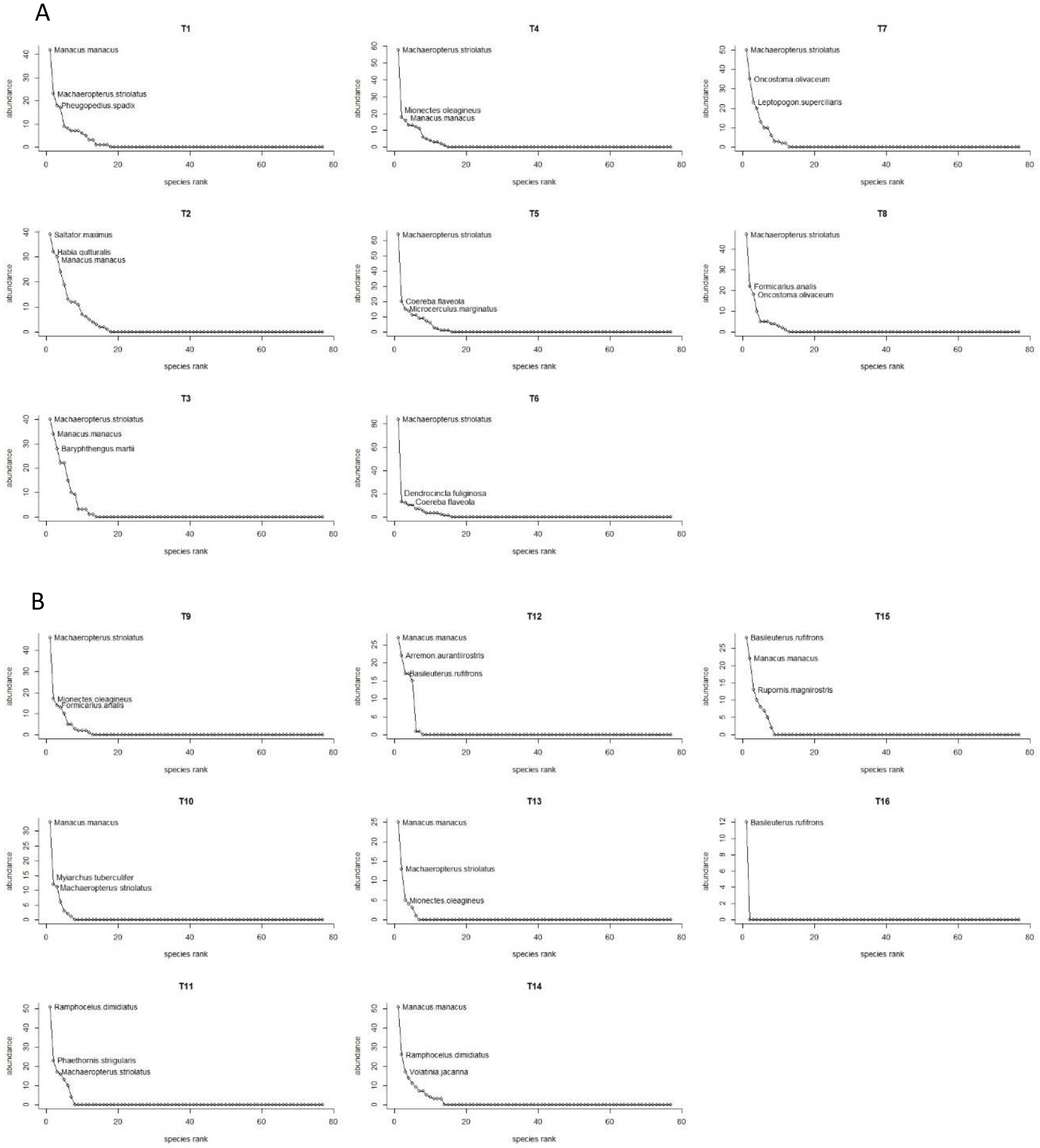

A
T1
Manacus.manacus
Machaeropterus.striolatus
Pheugopedius.spadix
T4
Machaeropterus.striolatus
Mionectes.oleagineus
Manacus.manacus
T7
Machaeropterus.striolatus
Oncostoma.olivaceum
Leptopogon.superciliaris
T2
Saltator.maximus
Habia.gutturalis
Manacus.manacus
T5
Machaeropterus.striolatus
Coereba.flaveola
Microcerculus.marginatus
T8
Machaeropterus.striolatus
Formicarius.analis
Oncostoma.olivaceum
T3
Machaeropterus.striolatus
Manacus.manacus
Baryphthengus.martii
T6
Machaeropterus.striolatus
Dendrocincla.fuliginosa
Coereba.flaveola
B
T9
Machaeropterus.striolatus
Mionectes.oleagineus
Formicarius.analis
T12
Manacus.manacus
Arremon.aurantiirostris
Basileuterus.rufifrons
T15
Basileuterus.rufifrons
Manacus.manacus
Rupornis.magnirostris
T10
Manacus.manacus
Myiarchus.tuberculifer
Machaeropterus.striolatus
T13
Manacus.manacus
Machaeropterus.striolatus
Mionectes.oleagineus
T16
Basileuterus.rufifrons
T11
Ramphocelus.dimidiatus
Phaethornis.striigularis
Machaeropterus.striolatus
T14
Manacus.manacus
Ramphocelus.dimidiatus
Volatinia.jacarina
abundance
species rank

C

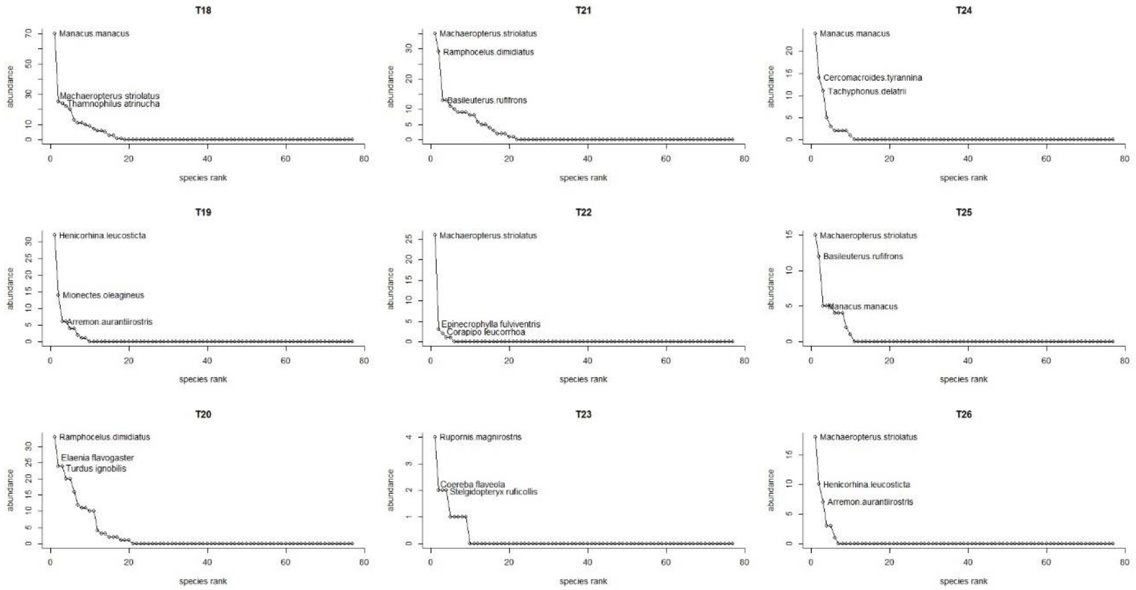

Supplementary Figure 1. Rank abundance curves in each transect for A. Playas, B. Porce II and C. Porce III.

The rank abundance curves for each evaluated transect are depicted in the figures. The y-axis represents the absolute abundance of each species, while the x-axis shows the cumulative number of species detected at each site, based on the subsample of species captured in mist-nets. In the figures, only the three most abundant species per site are displayed. Species such as *Manacus manacus, Machaeropterus striolatus* and *Arremon aurantiirostris* exhibit the highest abundances.

Supplementary Table 4. Point counts for record the bird species.

Dam	Code	Latitude	Longitude	Altitude	Coverage
Playas	T1P1-P	6.3493333	-74.9933055	1012	Bdatf
	T1P2-P	6.3490833	-74.9908333	1056	Bdatf
	T1P3-P	6.3333611	-74.9881111	1045	Bdatf
	T2P1-P	6.34097222	-74.9840833	1014	Bdatf
	T2P2-P	6.34383333	-74.9845278	1007	Bdatf
	T2P3-P	6.34658333	-74.9846111	1051	Pe
	T2P4-P	6.34705556	-74.9873889	1054	Pe
	T3P1-P	6.32638889	-74.9949444	1010	Bdatf
	T3P2-P	6.32805556	-74.9969722	1053	Bdatf
	T3P3-P	6.33063889	-74.9955	1078	Bdatf
	T3P4-P	6.33263889	-74.9976389	1146	Bdatf
	T4P1-P	6.31880556	-74.9521389	1062	Bdatf
	T4P2-P	6.32063889	-74.9537778	1121	Bdatf
	T4P3-P	6.32138889	-74.9563611	1112	Bdatf
	T4P4-P	6.32055556	-74.9583889	1059	Bdatf
	T5P1-P	6.29879	-74.92797	1189	Vsb
	T5P2-P	6.30142	-74.92904	1093	Bf
	T5P3-P	6.304043	-74.930207	1189	Bf
	T5P4-P	6.30605	-74.93095	1036	Bf
	T6P1-P	6.260235	-74.927222	1036	Bdatf
	T6P2-P	6.263804	-74.92718	1037	Bdatf
	T6P3-P	6.266957	-74.930101	1093	Bdatf
	T6P4-P	6.267885	-74.932718	1126	Bdatf
	T7P1-P	6.302276	-74.994427	1020	Bf
	T7P2-P	6.305217	-74.994241	1029	Bf
	T7P3-P	6.307858	-74.994769	1036	Bf
	T7P4-P	6.310356	-74.99524	1036	Bf
	T8P1-P	6.277384	-74.928	926	Bdatf
	T8P2-P	6.280539	-74.92947	1015	Bdatf
	T8P3-P	6.280939	-74.9361969	1103	Bdatf
	T8P4-P	6.283381	-74.934053	1090	Bdatf
Porce II	T1P1-PII	6.761731	-75.103136	999	Bdatf
	T1P2-PII	6.763001	-75.10073	1039	Bdatf
	T1P3-PII	6.764599	-75.09851	1082	Bdatf
	T2P1-PII	6.769562	-75.098666	1020	Vsa
	T2P2-PII	6.772176	-75.099515	1043	Vsa
	T2P3-PII	6.774075	-75.101543	1066	Vsa
	T3P1-PII	6.777441	-75.095468	986	Vsa
	T3P2-PII	6.775815	-75.093207	967	Vsa
	T3P3-PII	6.774787	-75.095887	999	Vsa
	T4P1-PII	6.795318	-75.120884	1006	Pe
	T4P2-PII	6.792377	-75.121247	991	Bdatf
	T4P3-PII	6.7902	-75.122909	974	Bdatf
	T5P1-PII	6.77485	-75.078943	933	Bdatf
	T5P2-PII	6.776856	-75.076933	933	Bdatf
	T5P3-PII	6.778054	-75.074432	1002	Bdatf
	T6P1-PII	6.779704	-75.083377	960	Pe
	T6P2-PII	6.782288	-75.08405	904	Vsb
	T6P3-PII	6.776919	-75.082831	935	Vsb
	T7P1-PII	6.796372	-75.129647	977	Vsa
	T7P2-PII	6.796342	-75.132417	939	Vsa

Dam	Code	Latitude	Longitude	Altitude	Coverage
	T7P3-PII	6.795544	-75.126502	987	Vsa
	T8P1-PII	6.777826	-75.115138	1008	Vsa
	T8P2-PII	6.779202	-75.113084	1006	Vsa
	T9P1-PII	6.781526	-75.122774	974	Bdatf
	T9P2-PII	6.779106	-75.123975	976	Bdatf
Porce III	T1P1-PIII	6.961062	-75.097603	542	Vsa
	T1P2-PIII	6.961602	-75.095307	486	Bdatf
	T1P3-PIII	6.962627	-75.092646	522	Bdatf
	T1P4-PIII	6.964034	-75.090343	565	Bdatf
	T2P1-PIII	6.981674	-75.101948	1358	Bdatf
	T2P2-PIII	6.978325	-75.097811	1251	Bdatf
	T2P3-PIII	6.978724	-75.092222	1099	Vsa
	T2P4-PIII	6.983872	-75.0901	899	Vsa
	T3P1-PIII	6.973653	-75.092095	925	Pe
	T3P2-PIII	6.970916	-75.093135	915	Pe
	T3P3-PIII	6.971341	-75.090556	864	Pe
	T4P1-PIII	6.997104	-75.07524	696	Vsa
	T4P2-PIII	6.999859	-75.075067	656	Vsa
	T4P3-PIII	6.995832	-75.077822	695	Vsa
	T5P1-PIII	6.89955	-75.150935	1462	Bdatf
	T5P2-PIII	6.901459	-75.153102	1449	Bdatf
	T5P3-PIII	6.901668	-75.155911	1367	Bdatf
	T6P1-PIII	7.027035	-75.038418	1062	Mefn
	T6P2-PIII	7.026841	-75.041213	1006	Mefn
	T6P3-PIII	7.026081	-75.043806	917	Mefn
	T7P1-PIII	7.02173	-75.058457	380	Vsb
	T7P2-PIII	7.023403	-75.06053	484	Vsa
	T7P3-PIII	7.019062	-75.0595	382	Vsa
	T8P1-PIII	6.913327	-75.165998	681	Emb
	T8P2-PIII	6.910629	-75.166483	776	Vsb
	T8P3-PIII	6.908277	-75.167317	818	Vsb
	T9P1-PIII	6.935361	-75.138518	658	Emb
	T9P2-PIII	6.936764	-75.136176	771	Bdatf
	T9P3-PIII	6.937579	-75.133558	832	Vsa

*Bdatf: High dense upland forest, Vsa: High secondary vegetation; Vsb: Low secundary vegetation, Bf: Fragmented forest, Pe: Weedy pastures, Mefn: Mix of native forest species, Emb: Dam.

In this table we shows the decimal coordinates taken in the field for each observation point. The code corresponds to each evaluated transect and each point counts in the different dams.

Supplementary Table 5. Procedure and reagents used in the molecular identification of Haemosporidian.

PCR	Reactives (Vol 10µl)	Thermal profile
Mitochoncdrial gene ND4	1 µl ADN + 1 µl dNTPs + 1 µl Taq buffer+ KCl + 0,8 µl MgCl2 + 0,5 µl ND4 + 0,5 µl LEU + 0,1 µl Taq Polimerasa + 5,1 µl ddH2O.	Denaturation at 95°C for 3 and 1 min, alignment at 60°C for 1 min, extension at 72°C for 1:10 min, followed by 34 cycles denaturation and a final extension of 72°C for 10 min
Universal for Haemosporidia (*cyt b*)	1 µl ADN + 1 µl dNTPs + 1 µl Taq buffer+ KCl + 0,8 µl MgCl2 + 0,5 µl HAEMF1 + 0,5 µl HAEMR3 + 0,15 µl Taq Polimerasa + 5,05 µl ddH2O.	Denaturation at 95°C for 3 and 0:20 min, alignment at 50°C for 0:30 min, extension at 72°C for 0:45 min, followed by 19 cycles denaturation and a final extension of 72°C for 10 min
Plasmodium/ *Haemoproteus*	1 µl ADN + 1 µl dNTPs + 1 µl Taq buffer+ KCl + 0,6 µl MgCl2 + 0,3 µl HAEMF + 0,3 µl HAEMR2 + 0,15 µl Taq Polimerasa + 5,65 µl de ddH2O.	Denaturation at 95°C for 3 y 0:30 min, alignment at 55°C for 0:45 min, extension at 72 °C for 0:45 min, followed by 39 cycles denaturation and a final extension of 72 °C for 10 min
Leucocytozoon	1 µl ADN + 1 µl dNTPs + 1 µl Taq buffer+ KCl + 0,8 µl MgCl2 + 0,5 µl HAEMFL + 0,5 µl HAEMR2L + 0,15 µl Taq Polimerasa + 5,05 µl ddH2O.	Denaturation at 95 °C for 3 and 0:30 min, alignment at 57 °C for 0:45 min, extension at 72 °C by 0:45 min, followed by 39 cycles denaturation and a final extension of 72 °C for 10 min

This table summarizes the reactives and protocols for each PCR performed for the diagnosis of avian haemosporidian. DNA quality was tested by the amplification of a fragment of the avian ND4 mitochondrial gene with primers ND4 and LEU[41]. Samples with good DNA quality (those that successfully amplified the avian ND4 fragment) were used for the molecular diagnosis of haemosporidian lineages of the genera *Plasmodium*, *Haemoproteus* and *Leucocytozoon*. Haemosporidian were screened using a nested PCR protocol to amplify a fragment of the parasite mitochondrial gene cyt-b. Briefly, a universal PCR was performed to amplify a fragment common to the three parasites in a first PCR reaction. A subsequent nested PCR was performed to separately amplify the parasites of the genera *Plasmodium* and *Haemoproteus*[42] and *Leucocytozoon*[43]. Each PCR was analyzed at least twice to avoid the occurrence of false negatives. Reactions included a positive control (sample with positive infection confirmed by sequencing and microscopy) and a negative control (reaction without DNA).
